# Behavioral Characterization of a Mouse Model Overexpressing *DSCR1/ RCAN1*

**DOI:** 10.1371/journal.pone.0017010

**Published:** 2011-02-25

**Authors:** Mara Dierssen, Gloria Arqué, Jerome McDonald, Nuria Andreu, Carmen Martínez-Cué, Jesús Flórez, Cristina Fillat

**Affiliations:** 1 Genes and Disease Program, Centre for Genomic Regulation (CRG), Barcelona Biomedical Research Park (PRBB), and CIBER de Enfermedades Raras (CIBERER), Barcelona, Catalonia, Spain; 2 Department of Physiology and Pharmacology, Faculty of Medicine, University of Cantabria, Santander, Spain; 3 Fundación Síndrome de Down de Cantabria, Fundación Iberoamericana Down21, Santander, Spain; Université Pierre et Marie Curie, France

## Abstract

*DSCR1/ RCAN1* is a chromosome 21 gene found to be overexpressed in the brains of Down syndrome (DS) and postulated as a good candidate to contribute to mental disability. However, even though *Rcan1* knockout mice have pronounced spatial learning and memory deficits, the possible deleterious effects of its overexpression in DS are not well understood. We have generated a transgenic mouse model overexpressing DSCR1/RCAN1 in the brain and analyzed the effect of RCAN1 overexpression on cognitive function. Tg*RCAN1* mice present a marked disruption of the learning process in a visuo-spatial learning task. However, no significant differences were observed in the performance of the memory phase of the test (removal session) nor in a step-down passive avoidance task, thus suggesting that once learning has been established, the animals are able to consolidate the information in the longer term.

## Introduction

Mental disability in individuals with Down syndrome (DS) is produced by the increased expression of some of the 231 supernumerary genes on the extra copy of chromosome 21. One such gene is Down's syndrome candidate region-1 (*DSCR1*, also known as *RCAN1*) [1], [2], which encodes a protein that is a functional inhibitor of calcineurin, a ubiquitous and multifunctional calcium-activated protein phosphatase. It is located in the Down syndrome critical region and its expression is consistently increased 1.8-fold in Down's syndrome human fetal tissues and in Ts65Dn mice [3].

RCAN1 physically and functionally interacts with calcineurin A, effectively inhibiting its phosphatase activity [4]. Calcineurin is the most abundant protein in brain where it regulates, among other neuronal functions, neurotransmitter release, neurite outgrowth and neuronal cell death. The only calcineurin inhibitor which expression is regulated by calcium-calcineurin signaling is RCAN1, indicating that this protein can function in a feedback inhibition loop to suppress sustained calcineurin activity. RCAN1 is widely expressed in brain, both during development and in the adult and it has been postulated as a good candidate to contribute to mental disability in DS [5], [6], [7]. Mouse models of RCAN1 overexpression have shown a role of RCAN1 regulating exocytosis in chromaffin cells and in mutant huntingtin phosphorilation, suggesting a role of RCAN1 in the Alzheimer's disease neuropathology associated to DS and in Huntington's disease [8], [9], [10]. Moreover, it has been identified as a gene involved in cognitive symptoms of schizophrenia patients by measuring differences in DNA copy number variants [11]. Evidences of an RCAN1 role in cognition also exist from studies in experimental models. *Drosophila* mutants with both loss-of-function or overexpression of nebula exhibit severe learning defects that are attributed by biochemical perturbations rather than maldevelopment of the brain [12] and the *Rcan1* knockout mice present pronounced spatial learning and memory deficits in the Morris water maze task and impaired long-term potentiation (LTP) [13]. However, the *in vivo* relevance of Rcan1 overexpression and its possible contribution to DS cognitive phenotypes has not yet been explored.

In the current study we have analyzed the effect of RCAN1 overexpression on the cognitive function using transgenic mice. We have focused on the potential role of this regulator in processes related to learning and memory in DS by characterizing the neurological and cognitive alterations in these mice. Our work confirms a role of RCAN1 overexpression in the visuo-spatial learning and memory tasks, with alterations similar to those present in DS persons.

## Materials and Methods

### Generation of TgRCAN1 transgenic mice

The transgenic cassette was generated by cloning a 1.3 Kb fragment of the human *PDGFβ* chain promoter in the 5′ region of a plasmid containing the second intron of the β-globin gene, flanked by part of exons 2 and 3, and the polyadenilation signal of the SV-40 virus. The 0.6 Kb *RCAN1* cDNA was introduced in the *EcoRI* site of the β-globin exon 3. The complete cassette was sequenced with specific primers in an automated sequencer (Applied Biosystems 373A) to ensure the integrity of the transgene. The *PDGFβ-RCAN1* construct was isolated from the plasmid sequences by digestion with *Xba*I and *Xho*I, gel purified using the GenClean spin kit (Q-Biogene) and microinjected (2–5 µg/ml) into B6/SJL zygotes, which were transferred to the oviducts of 0.5 dpc pseudopregnant CD1 females. Genotyping of founder mice was performed by Southern blotting. Genomic DNA was obtained from mice tail biopsy. Ten µg of DNA were digested with *EcoR*I, electrophoresed in 1% agarose and transferred to nitrocellulose membranes (Hybond-N+; Amersham Pharmacia Biotech). Hybridization of the filters was done according to manufacturer procedures (Amersham Pharmacia Biotech), using the human *RCAN1* cDNAs as a probe. Three transgenic lines were obtained and maintained in hemizygosity by crossing with B6/SJL mice. Transgene copy number was also determined by Southern blot analysis. Autoradiographies were analyzed using Phoretix 1D software (Nonlinear Dynamics Ltd, Newcastle Upon Tyne, UK). Genotyping of the progeny was performed by multiplex PCR analysis using the human specific primer pairs: *RCAN1*-F primer, 5′- AGGACGTATGACAAGGACAT -3′ and *RCAN1*-R primer, 5′-ATCAGAAACTGCTTGTCTGGA-3′; GdX-F primer, 5′- GGCAGCTGATCTCCAAAGTCCTGG-3′ and GdX-R primer, 5′- AACGTTCGATGTCATCCAGTGTTA -3′. PCR conditions were denaturation at 94°C for 15 sec, annealing at 56°C for 15 sec, and extension at 72°C for 30 sec, for 32 cycles.

### RT-PCR analysis

Total RNA was prepared from different brain regions (hippocampus, cerebral cortex and cerebellum) of Tg*RCAN1* and control mice using TriPure reagent (Roche). To avoid genomic contamination RNA samples were treated with DNAse (DNAfree, Ambion), as described by manufacturer's protocol. One µg of total RNA from each sample was reverse transcribed with a Retroscript RT kit (Ambion). Two µl of cDNA was PCR amplified with the same conditions described above for genotyping with the human specific primers (*RCAN1*-F and *RCAN1*-R). Absence of genomic DNA contamination was determined by the amplification of a 126 bp PCR fragment from cDNA samples with primers for GdX transcript (GdX-F and GdX-R).

### Western blot analysis

Western blot analysis from adult brain samples was performed as described by Porta et al. with minor modifications [5]. Briefly, individual samples were mechanically homogenized in a glass potter, with a lysis buffer, containing: 50 mM Tris-HCl pH 7.4, 10 mM EDTA, 320 mM sucrose, 1 mM phenylmethylsulfonyl fluoride and a protease inhibitor cocktail (Complete Mini, Roche Diagnostics, Mannheim, Germany). Extracts were centrifuged for 10 min at 800×g for 10 min at 4°C, and the protein concentration determined from the cleared lysate (BCA assay; Pierce, Rockford, IL). Proteins were separated by 12% SDS-PAGE gel and transferred onto nitrocellulose membranes (HybondTM-C, Amersham Biosciences, Freiburg, Germany). The membranes were blocked in 10% non-fat dried milk dissolved in TBS-T (10 mM Tris-HCl pH 7.5, 100 mM NaCl, 0,1% Tween 20) for 1 h at room temperature and then incubated overnight at 4°C with the primary antibody, a rabbit polyclonal anti-RCAN1 [5], diluted 1∶1000 in 5% powdered milk in TBS-T. Protein loading was monitored using a mouse antibody against α-tubulin (1∶5000, Sigma). Incubation with anti-rabbit or anti-mouse IgG/HRP (horseradish peroxidase) antibodies (1∶2000; Dako, Glostrup, Denmark) was performed at room temperature for 1 h. Membranes were rinsed in PBS-T and the immunocomplexes were detected by enhanced chemiluminiscence with ECL™ Western blotting detection reagent (Amersham Bioscience). Chemiluminiscence was determined with a LAS-3000 image analyzer (Fuji PhotoFilm Co., Carrollton, TX, USA).

### General histology

Mice were anaesthetized and then perfused transcardially with 0.1 M PBS, followed by chilled 4% paraformaldehyde in PBS. At least five animals were used per genotype and age. Brains were removed from the skull and postfixed in the same fixative for 24 h at 4°C overnight. After rinsing in PBS, brains of adult mice were cryoprotected in 30% sucrose and kept frozen at −80°C. Coronal sections 50 µm thick were cut with a cryostat and maintained in cryoprotective solution at −20°C until use. Sections were mounted in slices, air-dried, dehydrated in increasing concentrations of ethanol followed by xilene and coverslipped with DPX mounting medium. Cresyl violet staining was performed to determine the total cell population.

### Behavioral analysis

#### Animals

Transgenic mice were obtained in heterozygosity in B6/SJL-F1J genetic background. Hybrid founders were crossed using B6/SJL-F1J females and all experiments were performed using mice from the F1–F5 generation to attenuate littermate's genetic differences. We have used male mice in order to avoid female estrous cycle variations. The non-transgenic (WT) littermates obtained from crosses of males Tg*RCAN1* mice and B6/SJL-F1J females served as controls. Mice were housed in groups of 3–5 animals per cage in standard macrolon cages (40 cm long×25 cm wide×20 cm high) under a 12 h light/dark schedule (lights on 08:00 to 20:00) in controlled environmental conditions of humidity (50%–70%) and temperature (22±2°C) with food and water supplied *ad libitum*. All experimental procedures were approved by the local ethical committee (CEEA-IMIM and CEEA-PRBB), and met the guidelines of the local (Catalan law 5/1995 and Decrees 214/97, 32/2007) and European regulations (EU directives 86/609 and 2001-486) and the Standards for Use of Laboratory Animals n° A5388-01 (NIH).

#### Neurological assessment (SHIRPA protocol)

A comprehensive testing protocol was used to identify and characterize phenotype impairments as previously described [14], [15]. Assessment of each animal began with observation of undisturbed behavior in a cylindrical clear Perspex viewing jar (15 cm height×11 cm diameter) for wild running or stereotypy. The mice were then transferred to an arena (56×34 cm) for observation of motor behavior and sensorial function. Animals underwent screening exams for visual acuity, vibrissae, corneal and pinna responses to an approaching cotton swab, auditory function (Preyer reflex), vestibular function (contact righting reflex and negative geotaxis), and grip strength and body tone. In the last part of the test, changes in excitability, aggression, general fear, vocalization and salivation and piloerection (for analysis of autonomic function) were recorded.

#### Coat-hanger test

Motor coordination was initially measured using the coat-hanger test, placing the mice in the middle of the wire in an upside-down position. First, the prehensile reflex was evaluated and was rated as (0) if the mouse fell off the hanger and as (1) if it remained hanging during a 5 sec period. During this period traction capacity was rated as (0) if mice did not lifted-up their hind limbs, (1) if they lifted one of the hind limbs, (2) if they lifted both, and (3) if they reached one of the ends of the hanger. After this initial evaluation, the latency to fall, time to reach one of the ends of the hanger, as well as the activity performed on the wire was measured in a single trial lasting 60 sec.

#### Balance beam test

The ability of the mice to maintain balance was evaluated on a wooden bar (9 cm diameter×50 cm long×12 mm thick) during a period of 40 sec. Mice were placed with their forelimbs on the bar and two trials were performed. The score was rated as follows: (0) if the mouse fell before the 40 sec period; (1) if the mouse remained in the center of the bar; (2) if it moved out of the center, but it did not reach the ends; (3) if it reached one of the ends.

#### Open field

The open field was a white Plexiglas apparatus (70 cm wide×70 cm long×25 cm high) divided into 25 equal squares, it were delimited with black lines on the white floor of the box and under high intensity light levels (300 luxes). Two zones, the centre and periphery, were delineated being the centre more anxiogenic. At the beginning of the test session, mice were left in the middle of the apparatus and the latency to cross from the centre to the periphery, the distance traveled, velocity and time spent in each zone and rearing activity, defecation (number of fecal boluses) and grooming behavior were scored during 5 min.

#### Rotating rod test

To test motor coordination and balance, the ability of each mouse to maintain balance was assessed on a rotating rod (5 cm diameter×10 cm long) with a plastic dowel surface (Rotarod LE8500, Panlab SA, Barcelona, Spain). The equipment consisted of a rotating spindle that is able to maintain a fixed rotational speed (revolutions per min, rpm) and to undergo an acceleration cycle, starting at 4 rpm and accelerating at a constant rate to 40 rpm over a 1 min period. The apparatus is provided with magnetic plates to detect when a mouse has fallen off the rod. Mice were placed on the middle of the rotating rod, its body axis being perpendicular to the rotation axis, and its head against the direction of rotation. All animals were tested for acquisition and maintenance of rotating performance. The experimental design consisted of two training sessions (day 1 and 2) in which the number of trials required to learn to remain on the rod during 180 sec at the minimum speed (4 rpm) was recorded. After the mice reached the criterion, a third session (day 3) was carried out in which two different tasks were performed: motor coordination and balance were assessed (a) by measuring the latency to fall off the rod in consecutive trials with increasing fixed rotational speeds (7, 10, 14, 19, 24, and 34 rpm), and the animals were allowed to stay on the rod for a maximum period of 240 sec per trial and a resting period of 15 min was left between trials; and (b) for the accelerating rod test, the rotation speed was increased during a single session of 60 sec from 4 to 40 rpm. For each trial, the elapsed time until the mouse fell off the rod was recorded.

#### Visuo-spatial learning and memory in the water maze test

To test hippocampal-dependent spatial cognition, Tg*RCAN1* mice were trained in the standard Morris water maze (MWM) with a hidden platform as previously described [16]. The mice were tested over 6 days (4 trials/session, 10 min inter-trial intervals). The water maze consisted of a circular pool (diameter, 1.20 m; height, 0.5 m). It was filled with tepid water (24°C) opacified by the addition of powdered milk (0.9 kg). A white escape platform (15 cm diameter, height 24 cm) was located 1 cm below the water surface in a fixed position (NE quadrant, 22 cm away from the wall). In each trial, mice were placed at one of the starting locations in random order [north, south, east, west (N, S, E, W), including permutations of the four starting points per session] and were allowed to swim until they located the platform. Mice failing to find the platform within 60 sec were placed on it for 20 sec (the same period of time as the successful animals). At the end of every trial the mice were allowed to dry for 15 min in a heated enclosure and were returned to their home cage. The cue session was performed to test the swimming speed and visual ability using the visible platform, elevated 1 cm above the water and its position was clearly indicated by a visible cue (black flag). White curtains with affixed black patterns to provide an arrangement of spatial cues surrounded the maze. It was performed 24 hours after the fourth training sessions and 5 days after completion of the hidden platform training protocol. To test whether the mice remembered the location of the platform, probe trials were performed. In the probe session the platform was removed and mice were allowed to swim for 60 sec. The time spent in the trained and non-trained quadrants as well as the number of platform annulus crossings during 60 sec were recorded. On the next day (5 days after the last acquisition session), mice performed the reversal learning session. In this test, the platform position was changed to the opposite quadrant (SW).

All the trials were recorded and traced with an image tracking system (SMART, Panlab, Spain) connected to a video camera placed above the pool. Escape latencies, length of the swimming paths and swimming speed for each animal and trial were monitored and computed. The more in detail analysis of the performance of the mice, was performed using a custom-designed analysis program the jTracks software [17].

#### Passive Avoidance

We used a step-down passive avoidance test, which consisted of a transparent Plexiglas circular cage (40 cm in height, 30 cm in diameter) with a grid floor and a circular platform (4 cm diameter) in the center. During the training session, animals were placed on the platform and their latency to step down with all four paws was measured. Immediately after stepping down on the grid, animals received an electric shock (0.6 mA, 2 sec). Retention test sessions were carried out 24 h (short-term) and 7 days after training (long-term). Step-down latency was used as a measure of memory retention. A cut-off time of 300 sec was set.

### Data analysis

Simple comparisons between Tg*RCAN1* and control mice in various tasks were performed using the two-tailed unpaired Student *t* test with Mann-Withney's correction to account for the different variances in the populations being studied. Performances on the Rotarod were compared using repeated measures ANOVA. To assess significant differences in the behavior of both transgenic lines a one-way ANOVA with Bonferroni test for post hoc analyses was performed. In the Morris Water Maze a repeated measures ANOVA test was used. The passive avoidance test was analyzed using Mann-Withney U non-parametric test. Data were summarized as mean ± standard error of mean (S.E.M.). In all tests, a difference was considered to be significant if the obtained probability value was P<0.05. The statistical analysis was performed using the SPSS 12.0 software.

## Results

### Tg*RCAN1* mice are viable and show no gross abnormal phenotype

**Figure 1:**
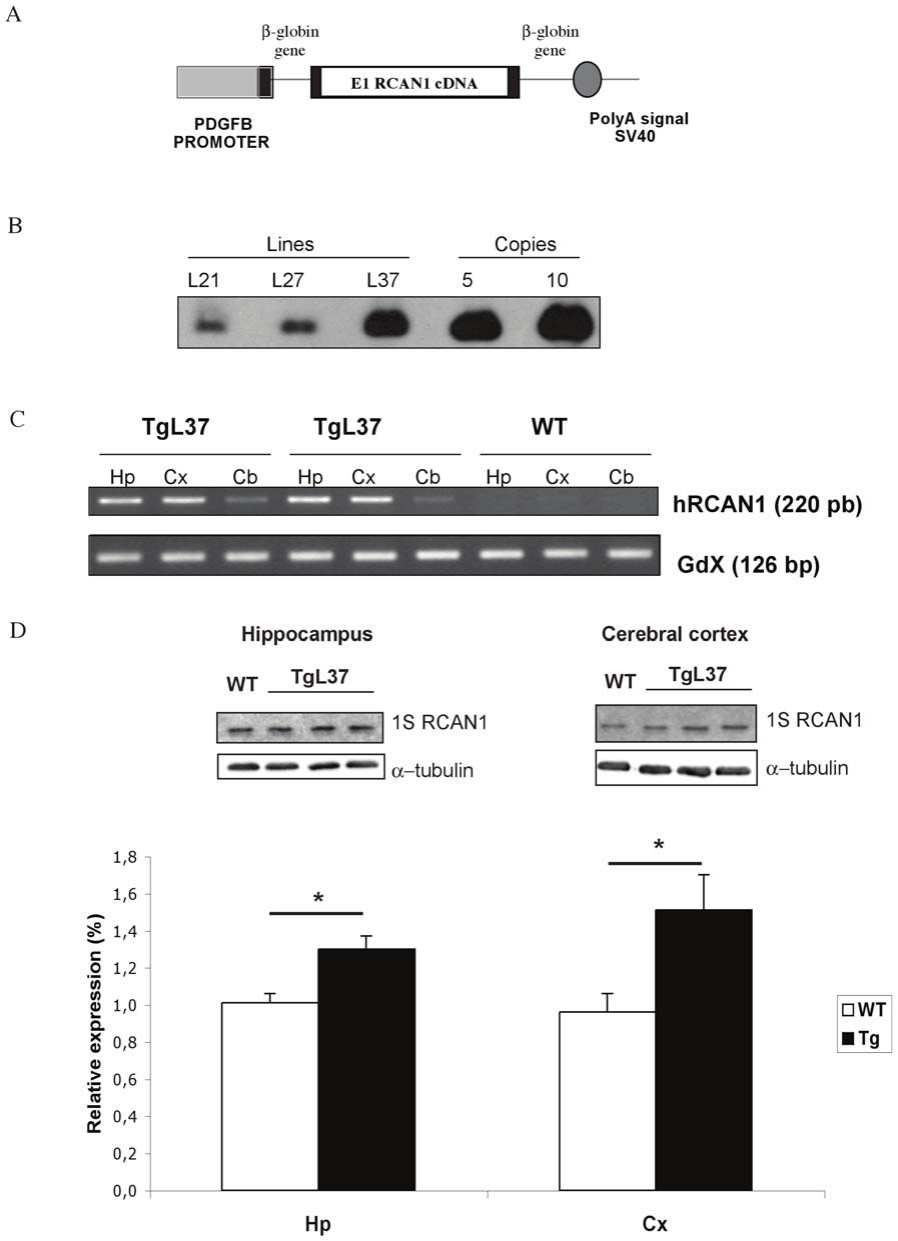
Genotyping and expression analysis of Tg*RCAN1* mice. **A**) Schematic representation of the *PDGFβ/RCAN1* chimeric gene. **B**) Southern blot analysis of three independent transgenic lines (L21, L27 and L37). **C**) Expression of the transgene by RT-PCR analysis in hippocampus (Hp), cerebral cortex (Cx) and cerebellum (Cb) of Tg*RCAN1* and control mice. **D**) Western blot analysis and relative quantification of RCAN1 protein levels from adult wild type (WT n = 6) and transgenic mice (TgL37 n = 8), in cerebral cortex and hippocampus. α-Tubulin was used as an internal loading control. Bars show densitometric analysis of Rcan1 normalized against the α-tubulin band (Hp: hippocampus, Cx: cerebral cortex). Data are represented as mean ± S.E.M. *P<0.05, Student's t test.

To generate the transgenic mice Tg*RCAN1*, the human isoform splice variant 1 was placed under the control of the PDGFβ promoter to drive transgene expression to brain areas [18], [19] **(** **Fig. 1A** **)**. Three transgenic lines were generated **(** **Fig. 1B** **)** that expressed the human RCAN1 isoform in the brain regions analyzed, being more abundant in the hippocampus and cerebral cortex as shown by RT-PCR **(** **Fig. 1C** **)**. No expression of hRCAN1 was detected in WT mice. Western blot analysis confirmed RCAN1 overexpression that was estimated in 1.3-fold and 1.5-fold in the hippocampus and cerebral cortex respectively **(** **Fig. 1D** **)**. No differences among transgenic lines and between genotypes were observed in terms of viability. Neurological assesment using the comprehensive SHIRPA protocol did not reveal any differences between Tg*RCAN1* and the control littermates, suggesting no gross impairment in general neural function. The observational assessment of behaviour in basal conditions showed no changes in excitability, fear, or aggressive behaviour **(** **Table 1** **)**. General histological staining did not reveal gross abnormalities in Tg*RCAN1* brains.

**Table 1:** SHIRPA primary behavioral screen.

	WT	TgRCAN1	P
Piloerection			
Absent	60%	75%	NS
Present	40%	25%	NS
Tail Elevation			
Horizontally extended	80%	100%	NS
Elevated /Straub tail	20%	0%	NS
Negative Geotaxis			
Turns and climbs the grid	100%	100%	NS
Touch escape			
Escape response to a firm stroke	20%	25%	NS
Escape response to a light stroke	80%	75%	NS
Trunk curling			
Absent	100%	100%	NS
Visual Placing			
Extension of forelimbs before contact	100%	100%	NS
Preyer Reflex			
Absent	20%	12,5%	NS
Flick of pinnae	60%	87,5%	NS
Startle	20%	0%	NS
Pinna Reflex			
Absent	40%	37,5%	NS
Active retraction	40%	62,5%	NS
Hyperactive, repetitive flicking	20%	0%	NS
Corneal Reflex			
Absent	10%	10%	NS
Active blink	90%	90%	NS
Provoked biting (absent)	100%	100%	NS
Righting Reflex (present)	100%	100%	NS
Toe Pinch			
Moderate withdrawal	80%	100%	NS
Brisk, rapid withdrawal	20%	0%	NS

Data are given as percentage of animals showing a specific phenotype. NS: non significant.

### Sensorimotor evaluation

**Figure 2:**
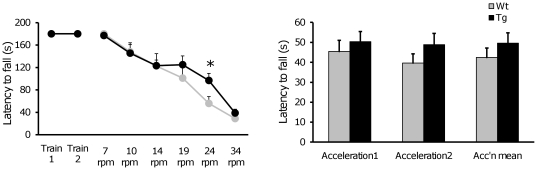
Motor learning and coordination in the Rotarod test. **Left panel**: Motor learning curve of Tg*RCAN1* and wild type mice. Tg*RCAN1* did not show impairment in motor learning but when submitted to consecutive trials at increasing rotational speeds (7, 10, 14, 24 and 34 rpm) performance in the more demanding trials was slightly improved in transgenic vs. wild type mice. **Right panel**: Acceleration trials. No differences were detected between genotypes. Each data point represents the mean ± S.E.M. *P≤0.05. Abbreviations: NS: non significant; rpm: revolutions per min.

Tg*RCAN1* mice did not show differences in the performance of a battery of sensorimotor tasks. No significant differences were attained in traction capacity (χ^2^ Pearson = 3.364, P = 0.762, data not shown), or when measuring the prehensile reflex, as shown by the similar latencies to fall in the 5 sec trial (F_(1, 54)_ = 0.866, P = 0.356, ANOVA). Similarly, in the balance beam test, the latencies to fall during 40 sec did not differ between genotypes (F_(1, 54)_ = 0.006, P = 0.938, ANOVA) thus suggesting that neither balance nor muscular strength were affected by RCAN1 overexpression. Also, no genotype-dependent differences were observed in the execution of the rotarod task (**Fig. 2**), neither during the training sessions (T1, T2), nor in the constant rotational speed sessions at low rpm (F_(1, 10)_ = 2.296, P = 0.161, MANOVA). However, at higher speed Tg*RCAN1* performed significantly worse than wild types (latency to fall from the rod at 24 rpm F_(1, 10)_ = 5.111, P = 0.0473, ANOVA). However, this may not be dependent on motor coordination since the more demanding acceleration trials showed no differences between genotypes (F_(1, 11)_ = 0.915, P = 0.359, ANOVA).

### Open Field

In the open field, Tg*RCAN1* mice showed a tendency to hyperactivity with increased distance travelled in the periphery (F_(1, 11)_ = 3.550, P = 0.086, ANOVA, data not shown), along with an increase in the mean speed found in Tg*RCAN1* mice (F_(1, 11)_ = 2.544, P = 0.139, ANOVA) This phenotype probably reflects a higher anxiety level since the mice showed no increase in the distance traveled in the center.

### Morris Water Maze

**Figure 3:**
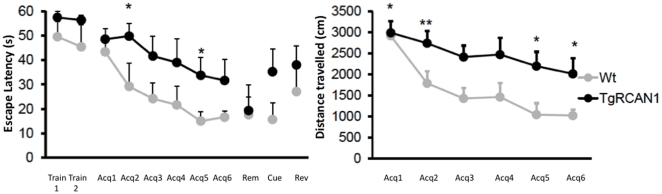
Visuo-spatial learning in the Morris water maze. Morris water maze performance of Tg*RCAN1* and wild type animals during the learning sessions expressed as (**left panel**) latency (s) to reach the platform along the acquisition phase, cue and reversal sessions. A clear deficit was observed in Tg*RCAN1* in all learning phases; (**Right panel**) Cumulative search-error on training trials and a learning index (Gallagher's proximity index) computed from the trials over the course of training. This measure relies on a computation of distance from the platform during the trial [17] and clearly indicated the use of poorer learning strategies in transgenic mice. White squares represent wild types and black squares represent Tg*RCAN1*. Data are represented as mean ± S.E.M. *P<0.05, **P<0.01.

We used the standard paradigm of the Morris water maze to analyze the visuo-spatial learning and memory profile in Tg*RCAN1* mice (**Fig. 3**). No genotype-related differences were observed in the training session, as demonstrated by the similar escape latency (ANOVA, F_(1,25)_ = 2.96; P = 0.87) and distance travelled (data not shown), although a tendency to perform worse the procedural learning task was detected in transgenic mice. Along the acquisition sessions, all groups of animals were efficient in learning the location of the platform as indicated by the progressive decrease in escape latency (repeated measures ANOVA, wild type, F_(1,23)_ = 100.7, P = 0.0001; Tg*RCAN1*, F_(1,16)_ = 110.5, P = 0.0001) and distance travelled (repeated measures ANOVA, P<0.001). However, an important learning impairment was detected in Tg*RCAN1* (repeated measures ANOVA, “session×genotype”, F_(1,28)_ = 14.11, P<0.001) mice as shown by the increased latencies (**Fig. 3**, left panel) to reach the platform and distances (repeated measures ANOVA, “session×genotype”, F_(1,28)_ = 16.14, P<0.001) **Fig. 3**, right panel) travelled across acquisition sessions. This was specifically related to learning problems since swimming speed was not affected and in the cued session, where the goal was to find a visible platform (black stripped flag), no differences were detected in the latency to reach the platform when it was made visible (ANOVA, F_(1,40)_ = 1.08; P = 0.31) suggesting that RCAN1 overexpression did not produce significant motor or motivational problems. During the probe trial that is a measure of the visuo-spatial memory, the latency to cross the annulus of the hidden escape platform and the number of crosses was similar between genotypes thus suggesting that once learned the information is retained in transgenic mice. Moreover, in both groups we observed a significantly higher preference for the trained quadrant (northwest), as shown by the percentage of time spent in the trained quadrant (ANOVA, wild-type, F_(1,23)_ = 58.6, P = 0.001 and Tg*RCAN1*, F_(1,16)_ = 56.7, P = 0.001). These observations support the conclusion that Tg*RCAN1* mice indeed remember the location of the platform. Finally, in the reversal session the efficiency to unlearn the old platform position and learn a new one was not altered in Tg*RCAN1*.

### Passive avoidance test

To further explore the learning and memory phenotypes, we used a step down passive avoidance paradigm. Retention memory was not altered either 24 hours or one week after training in transgenic mice, both genotypes showing increased retention latencies. This indicates that memory consolidation had taken place (data not shown).

## Discussion

Efforts to identify genes on human chromosome 21 with the potential to cause the brain anomalies observed in Down syndrome (DS), led to the discovery of a gene, Down syndrome candidate region-1 (*DSCR1*) now renamed *RCAN1* (Regulator of Calcineurin-1). RCAN1 is a modulator of calcineurin, a protein phosphatase known to function in a variety of cellular processes, among which learning and memory are relevant for the mental impairment seen in DS. Specifically, Hoeffer et al [13] reported that *Rcan1* knockout mice have pronounced spatial learning and memory deficits in the Morris water maze task, along with significant deficits in long-term potentiation (LTP) in the hippocampal area CA1, similar to what was found in mice with inducible, hippocampal-restricted overexpression of constitutively active calcineurin [20]. In animals in which calcineurin was inhibited by either transgenic expression of a calcineurin inhibitory domain or application of antisense oligonucleotides [21], [22] the opposite phenotypes were detected, thus suggesting that RCAN1 provides a constraint on calcineurin activity during learning and memory.

However, the threshold for hippocampal-dependent synaptic plasticity and memory storage seems to be determined by the balance between protein phosphorylation and dephosphorylation mediated by the kinase PKA and the phosphatase calcineurin. To establish whether an excessive inhibitory constraint on endogenous calcineurin in this balance may have a deleterious impact on learning and memory in DS, we examined the effect of genetically overexperessing *RCAN1*, and we have applied well-established mouse behavioral tests addressing hippocampal function. Moreover, we also explored other learning and memory processes, such as the recent memory, as tested in the passive avoidance paradigm.

Our data revealed neither motor nor sensory impairment in Tg*RCAN1* or changes in any of the neurological parameters tested. However, transgenic mice showed higher levels of activity in the open field similar to what was has been described in other DS models, such as Ts65Dn [16], [23] and in DS persons. This hyperactivity was not related to changes in emotionality, as it did not affect the activity in the central part of the field.

However, a marked phenotype was attained in the visuo-spatial learning paradigm. Although procedural learning was not affected in the pre-training session, the acquisition of the allocentric learning was markedly impaired in transgenic mice, thus suggesting that RCAN1 overexpression is specifically affecting hippocampal function. Those learning defects are specific since no differences were observed in swimming speed or in the motivational/motor aspects in the cued session. Moreover, the Gallagher index was markedly altered indicating the use of non-spatial learning strategies, and thus reinforcing the concept that RCAN1 overexpression leads to a specific and marked learning deficit in mice. However, in our experiments, Tg*RCAN1* mice did not show any impairment in the removal session, thus indicating that once consolidation has taken place, memory is well established. The poorer execution of transgenic mice during the acquisition phase was correlated with the searching trajectory of the mice, that was significantly different, so that wild type mice develop a clear spatial preference, whereas transgenic mice distribute their activity similarly across all quadrants, indicating impaired spatial learning.

Since the training procedure we have used involves an extensive acquisition phase, we also tested recent memory in a one-trial passive avoidance test to specifically targeting memory [24]. Again Tg*RCAN1* mice did not show any impairment reinforcing the concept that RCAN1 overexpression leads to specific alterations in the acquisition process but does not influence memory.

In conclusion, our results suggest that RCAN1 may contribute to specific learning phenotypes in DS patients. We also propose that excess of inhibition of endogenous calcineurin, which in turn acts as an inhibitory constraint in the hippocampal phosphorilation/dephosphorilation balance, has a significant impact on learning but not on memory.
